# Integrative Analysis Reveals the Prognostic Effects of Epigenetic Regulators in Bladder Cancer

**DOI:** 10.1002/cam4.71057

**Published:** 2025-07-22

**Authors:** Venugopalareddy Mekala, Yupei Lin, Xiang Wang, Naail Chowdhury, Jianrong Li, Chao Cheng

**Affiliations:** ^1^ Department of Medicine Baylor College of Medicine Houston Texas USA; ^2^ Dan L. Duncan Comprehensive Cancer Center, Baylor College of Medicine Houston Texas USA; ^3^ The Institute for Clinical and Translational Research, Baylor College of Medicine Houston Texas USA

**Keywords:** bladder cancer, epigenetic regulator genes, gene signatures, genomic aberrations, immune infiltration, prognostic prediction

## Abstract

**Background:**

Epigenetic regulatory genes (epiRG) are pivotal in the epigenetic regulation of the human genome, primarily through DNA and histone modifications. These genes are frequently mutated in human cancers, particularly bladder cancer (BC). However, the functional impact of epiRG mutations on patient outcomes remains poorly understood.

**Methods:**

In this study, we developed gene signatures for the most frequent genomic aberrations of epiRG using The Cancer Genome Atlas Bladder Carcinoma (TCGA‐BLCA) dataset and validated these signatures with independent tumor expression profiles for prognostic relevance. Furthermore, we evaluated the role of these signature scores in the immune system within the tumor microenvironment (TME). Finally, we assessed the correlation between epiRG and global DNA methylation.

**Results:**

Our results indicated that the inferred aberration‐specific signature scores were more predictive of patient stratification than the genomic aberrations. Notably, certain signature scores were significantly associated with patient progression, whereas others correlated with the tumor immune microenvironment via interactions with the immune system. Patients with mutations had high signature scores in CREBBP‐mut and EP300‐mut, which revealed poor overall survival. Conversely, KDM6A‐mut signatures showed an opposite trend, with low scores linking to favorable prognosis through enhanced immune activity. Also, other epiRG signature scores were strongly correlated with the immune system in TME and successfully predicted patients who responded to immunotherapy. Global methylation analysis revealed that high signature scores of KDM6A‐mut are associated with hypomethylation.

**Conclusions:**

These findings collectively establish epiRG signature scores as powerful biomarkers that integrate genomic, epigenetic, and immune microenvironment features for improved prognostic prediction in bladder cancer. This integrative approach not only advances our understanding of epigenetic mechanisms in BC but also offers potential for developing innovative prognostic tools and therapeutic strategies tailored to personalized medicine.

## Graphical abstract

Epigenetic regulatory gene (epiRG) mutations influence bladder cancer outcomes. Using TCGA‐BLCA data, we developed mutation‐specific gene signatures that predict prognosis better than mutations alone. These signatures correlate with immune infiltration and good survival outcomes.

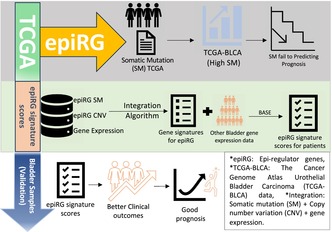

**Abbreviations**
AUC: area under the ROC curves
BC: bladder cancer
BCR: B‐cell receptor
BH: Benjamini‐Hochberg
CCL: chemokine ligand
CNV: copy number variations
COSMIC: Catalog of Somatic Mutations in Cancer
CpG: cytosines (C) followed by guanine residues (G) using one phosphate (p) group sequence
epiR: epigenetic regulators
epiRG: Epigenetic regulatory genes
FDR: false discovery rates
GEO: Gene Expression Omnibus
HAT: histone acetyltransferase
HR: Hazard Ratio
KM: Kaplan–Meier
MIBC: muscle‐invasive bladder cancer
NMIBC: non‐muscle invasive bladder cancer
OS: overall survival
SCC‐like: squamous cell carcinoma‐like
SM: somatic mutation's
TCGA‐BLCA: The Cancer Genome Atlas Bladder Carcinoma
TCGA: The Cancer Genome Atlas
TCR: T‐cell receptor
TFs: transcription factors
TIMER: Tumor Immune Estimation Resource
TME: tumor microenvironment
WT: wild‐type

## 1 Introduction

Epigenetic dysregulation is increasingly recognized as a significant factor in carcinogenesis. By strategically targeting these epigenetic regulators (epiR), scientists have been able to achieve improved clinical outcomes [1]. Proper epigenetic regulation is essential for maintaining normal cellular homeostasis; its deregulation can lead to tumor formation. These regulators, which include DNA methyltransferases, chromatin remodeling complexes, histone modification enzymes, and RNA‐mediated mechanisms, control gene expression without altering the DNA sequence across various cell types and developmental stages in cancer [1, 2, 3]. These functions influence cell differentiation, growth, and apoptosis, driving cells from a normal state toward a neoplastic state [4, 5, 6]. Consequently, genes carrying these functions are known as epiR genes (epiRG), opening new avenues for understanding and treating complex malignancies [7]. Recently, targeting epigenetic changes has shown promise in improving clinical outcomes across various diseases, including cancer [8, 9]. Epigenetic regulation represents a promising therapeutic avenue, supported by growing insights into epigenetic abnormalities in cancer [10]. However, genomic alterations in epigenetic regulator genes (epiRGs) are highly patient‐specific, limiting the ability to predict prognosis based solely on these aberrations [11]. Moreover, the functional consequences of many epiRG aberrations remain poorly understood, as highlighted in the Catalog of Somatic Mutations in Cancer (COSMIC) database [12]. As a result, understanding how these aberrations affect patient outcomes remains a significant challenge. There is a pressing need for alternative approaches that can better explain prognosis and guide therapeutic decision‐making.

BC, in particular, has a remarkably high rate of somatic mutations (SM) in epiRGs compared to other cancers [13]. This lethal and complex malignancy has exhibited alarming incidence rates, with 82,290 new cases and 16,710 deaths recorded in the USA in 2023 alone [14]. Aggressive alterations in epiRGs are major contributors to poor survival rates in BC, whereas clinical applications for prognostic stratification remain under development [5]. Targeting these aberrations represents a promising therapeutic strategy for improving clinical outcomes in BC, as these alterations are reversible [9].

Recent research has illustrated that chromatin modifier mutations were observed in epiRG, such as *CREBBP*, *EP300*, *ARID1A*, and *KDM6A*, and play a vital role in BC [13]. However, only mutations in *EP300* were associated with a favorable prognosis, leaving the prognostic value of other epiRG mutations unexplained in both The Cancer Genome Atlas (TCGA) and International Cancer Genome Consortium (ICGC) bladder patients [15]. *KDM6A*, a tumor suppressor gene, influences cancer cell growth. Its reduced expression caused by mutation or loss promotes cancer cell proliferation and inactivates downstream pathways. *KDM6A* interacts with genes involved in transcription factors (TFs), chromatin‐modifying enzymes, and the chromatin‐remodeling complex [16, 17]. In addition to individual epiRG implicated in BC, CREBBP and EP300 are critical components of the histone acetyltransferase (HAT) complex, which regulates core histone acetylation and plays a pivotal role in chromatin remodeling and transcriptional control. Both genes are frequently mutated in BC [11, 18]. Given their functional redundancy and cooperative activity, pharmacological targeting of the HAT complex has emerged as a promising therapeutic strategy in cancer treatment [19]. Studying both genes concurrently may also uncover shared cancer pathways and enable more comprehensive treatment approaches.

Meanwhile, genes with only copy number variations (CNV) show promise for BC prognosis. For example, one study demonstrated that amplification of the epiRG gene PRDM9 directly drives cancer progression. However, the prognostic significance of most other epiRG mutations remains unclear [20, 21]. These findings suggest that many factors beyond aberrations alone influence the complexity of carcinogenesis, including cellular genetics, abnormal cell division, and various oncogenic pathways that regulate alternative mechanisms. These aberrations have emerged as primary therapeutic targets, necessitating further research to develop alternative scores for patients [9]. Integrating gene expression profiles with genomic aberrations provides a comprehensive understanding of downstream pathway activity, thereby suppressor gene with the highest SM rate in many cancer types, including BC (48.8%). Evelien et al. found that compared to mutation status, the *P53* deficiency gene signature derived from the integration of gene expression accurately predicted drug sensitivity and overall survival (OS) in BC; we considered this as the benchmark for comparison with our results in this study [22]. Other studies have successfully demonstrated that signature scores for gene level proposed to predict BC patient prognosis [23, 24, 25]. No signature scores have been developed to elucidate the biological significance of epiRG aberrations in BC. The development of epiRG aberration signatures has emerged as a powerful tool for predicting clinical outcomes, particularly in cases where epigenetic alterations remain unclear [26, 27, 28]. This is the first study to demonstrate the clinical impact of epiRG aberrations in BC.

Considering these aspects, this study explores the concept of utilizing specific gene expression patterns as signatures to identify key genomic alterations driving BC development. We defined gene signatures exclusively based on 13 driver epiRG aberrations comprising 6 SM, 3 amplifications, and 4 deletions by integrating somatic mutation, CNV, and transcriptomic data from TCGA‐BLCA. We applied these signatures to multiple independent BC gene expression datasets, revealing the impact of dynamic changes and accurately stratifying patients based on signature scores and prognosis risk predictions. Moreover, we investigated the involvement of these signatures in cell proliferation and their interactions with immune infiltration in the tumor microenvironment (TME). Numerous studies have highlighted the critical role of immune cells in cancer initiation, progression, and the maintenance of a stable microenvironment [29, 30, 31]. Epi‐gene alterations can remodel the TME, making them prime targets for cancer therapies [32]. To understand this, we performed a correlation analysis to examine the association between aberration signature scores and the TME based on gene expression data. Factors, such as tumor cell proliferation, B‐cell receptor (BCR) richness (i.e., the diversity of unique BCR clonotypes), TGF‐beta response, macrophage abundance, leukocyte and stromal fractions, lymphocyte infiltration, and T‐cell receptor (TCR) richness all play crucial roles in either promoting or preventing cancer development within the TME [33]. For example, tumor cell proliferation is closely associated with aggressive cancer phenotypes and poor prognosis in BC, whereas TGF‐beta response is a key mediator of immune evasion and metastasis [34]. Similarly, BCR and TCR richness are indicators of the immune system's capacity to recognize and respond to cancer, with altered immune infiltrates correlating with patient outcomes in BC [35]. Notably, we also examined the correlation between these signatures and DNA methylation changes, which are essential for cell type differentiation and development [36]. Our study provides a framework to understand the genomic changes in epigenetic regulator genes underlying BC progression, holding promise for predicting clinical outcomes.

## 2 Methods

### 2.1 Overview of the Datasets Used in This Study

A pivotal finding from large‐scale cancer genome sequencing studies, as reported by Andrew P. et al. (2016), is the identification of recurrent mutations in 52 epigenetic modifier genes [6]. Importantly, the emergence of epigenetics‐modifying drugs in both preclinical and clinical settings has shown considerable promise, marking the beginning of an era of epigenetic‐oriented therapeutic strategies [10, 37, 38]. In the present study, these 52 genes serve as the primary resource for investigating the epigenetic landscape and therapeutic potential in cancer. To explore the mutational landscape of these genes, we retrieved SM data across 33 cancer types from the TCGA project via FireHose (http://firebrowse.org/). For SM data, we counted the total number of mutations for each gene corresponding to each sample and computed the mutation rate for each epiRG within every cancer type. Initially, we extracted the SM count data for all epiRG across all cancer patients. The processing of SM data (396 samples) involved three steps: (i) determining the count of mutations for each gene in all cancer patients for each specific cancer type, (ii) calculating the fraction of mutations for each gene within each cancer type, and (iii) calculating the total mutation rate for each gene across all cancer types. Based on preliminary results, we also gathered the clinical information like age, sex, survival days, event, etc. (412 samples), CNV (408 samples), gene expression data (408 samples: RSEM values) and DNA methylation data of Human Methylation 450 k contains cytosines (C) followed by guanine residues (G) using one phosphate (p) group sequence (CpG); these CpG levels contain beta values for each TCGA‐BLCA sample. Subsequently, in TCGA‐BLCA, CNV data for each gene is represented by segmented mean values. A positive segment mean indicates gene amplification or gain (fold increase > log2 (2.8/2)), whereas a negative segment mean denotes gene deletion or loss (fold decrease < log2 (1.4/2)). Copy number gains of tumor suppressor genes and losses of oncogenes generate common driver mutations in cancer [39]. Furthermore, we counted the number of amplifications and deletions for each epiRG and combined them with the epiRG BC mutation data.

Additionally, we observed a co‐occurrence of mutations in the CREBBP and EP300 genes using the cBioPortal platform (https://www.cbioportal.org/), where we selected the BC (TCGA, Cell 2017) option with these two genes and samples with both mutation and CNA data (408 samples/patients). Whether we checked how many samples have the mutually exclusive co‐mutations with the HAT complex. The statistical analysis is based on a Fisher's exact test performed within cBioPortal.

Additionally, publicly available BC patient datasets from the Gene Expression Omnibus (GEO) database were gathered for investigation under the accession IDs GSE48075 [40], GSE32894 [41], and GSE13507 [42]. The primary BC Choi dataset (GSE48075) was selected for the investigation of SM, consisting of 142 tumor samples of gene expression data and SM status for four genes FGFR3 (12‐mut/54‐wild), RB1 (7/59), PIK3CA (23/43), and TP53 (23/43). From the GSE32894 BC dataset, we gathered the 308 gene expression profiles and clinical information for 224 samples, which included molecular subtypes (111 urobasal‐A, 31 infiltrated, 56 genomically unstable, 12 squamous cell carcinoma‐like (SCC‐like), and 14 urobasal‐B samples), tumor stage, gender, time, and event data. In the GSE13507 dataset, out of 256 RNA‐seq data, 165 primary BC samples categorized into invasiveness (62 samples) and non‐muscle invasiveness (103 samples) alongside clinical information, such as TNM stage, age, overall survival time, and event were gathered. We transformed probe set expression into gene expression across all GEO datasets. In cases where genes had multiple probe sets, we selected the probe set with the highest average intensity across all samples to represent the respective genes.

### 2.2 Defining Gene Signatures for Each epiRG Aberration Based on TCGA‐BLCA Data

For the generation of gene signatures, we adhered to the methodology outlined by Zhao et al. [43]. Using this method, we defined the gene aberration‐weighted expression profiles [44] and excluded genes with expression values of 0 in ≥ 10 TCGA‐BLCA samples. We normalized the gene expression levels throughout the sample by transforming log10 (RSEM +1) values. For each gene, we calculated the univariate scores according to the gene expression fit into a linear model. For each patient, we related patient aberration status (*X* = 1 for mut/amp/del, *X* = 0 for WT) to gene expression. However, we have also calculated signatures using multivariate analysis after adjusting for age, gender, stage, and smoking status, but the outcome was not a better predictor for BC samples. For all genes, beta coefficients and statistical significance (*β* and *p*‐values) were estimated through linear regression. Using regression parameters, we defined weighted gene signature profiles for each epiRG, including positive weights (W^+^) assigned as −Log_10_(*p*‐value) for genes with *β* > 0, whereas negative weights (W^−^) were assigned as Log_10_(*p*‐value) for genes with *β* < 0. We further trimmed weights at 10 and transformed them into values within [0,1] by subtracting the minimum value and dividing by the range. Genes more significantly up‐regulated in aberration samples versus wild‐type (WT) samples were associated with higher (W^+^) compared to lower (W^−^) values, and vice versa for down‐regulated genes. We used the Choi dataset as a test set to evaluate these signature scores. Then, the predictive power of these signatures was further demonstrated through an ROC curve analysis, where the area under the ROC curve was plotted with sensitivities and specificities.

### 2.3 Calculation of Gene Signatures for Driver epiRG

The epiRG signature profiles in all genes were utilized to assess the enrichment of these signatures in patient samples. We employed the rank‐based gene set enrichment method BASE algorithm for datasets used in this study [45]. In the BASE algorithm, we used weighted profile regression results for all genes for epiRG aberrations and other gene expression data in the form of log_10_(gene expression + 1) as an input, using 1000 permutations and median normalization. We then calculated both up‐and down‐regulated profiles of epiRG aberration signature scores for each patient based on aberration status [44]. For other transcriptomic datasets, BASE assigns signature scores. A positive score indicates high activity in patient samples and high expression in the ranked gene list, whereas a negative score implies low activity and low expression in the ranked gene lists due to aberration changes.

### 2.4 Prediction of Patient Survival Using Driver epiRG Scores

For survival analysis, we used samples from the GSE32894 dataset to calculate epiRG signature scores and converted them to Z‐scores to maintain uniformity. We then performed univariate Cox proportional hazards models across all patients with an independent evaluation, focused on the group with the highest number of patients with overall survival (OS) rates. The Benjamini‐Hochberg (BH) method was employed to calculate false discovery rates (FDR). Four highly significant epiRG scores were selected for the construction of Kaplan–Meier (KM) curves. For KM plots, patients were stratified into two groups based on average signature scores as a cutoff (high scores = 1 and low scores = 0) and estimated Log‐rank test *p*‐values for each driver epiRG score. Additionally, we validated highly significant epiRG scores with the GSE13507 dataset, which had the second highest number of patients with OS information. Finally, we expanded our analysis to evaluate the distribution of signature scores for predicting improved clinical outcomes across both datasets.

Next, we also applied these signature scores to cell proliferation and immune infiltration in TME and global DNA methylation changes. For DNA methylation data, we computed *t*‐scores for each CpG (High *t*‐score > 0, Low *t*‐score < 0, and adjusted *p*‐value < 0.01) to identify up‐and down‐regulated CpGs for each epiRG aberration. The full details of data collection, preprocessing, and analysis can be found in Methods S1 section.

### 2.5 Statistical Analysis

In our exploration of changes of epiRG aberration, all statistical analyses were conducted using R software. Data normalization was consistently performed using log‐transformation. Student's *t*‐tests and Wilcoxon rank sum tests were performed to identify significant relations between two groups using “t.test” and “wilcox.test” functions. Cox proportional hazard models were created with the “coxph” function; for survival analysis, the “survival” function was implemented, and KM‐plots were generated using the “survminer” function. Spearman's rank correlation was conducted using the “cor” function to compute pairwise correlations, and *p*‐values were calculated using the “cor.test” function. Plots were constructed using “ggplot2”, “repel” and “ggpubr” R packages. Our analyses consistently applied a significance threshold of BH adjusted *p*‐value < 0.05. We used the statistical significance *p*‐value label as “ns” (not significant) for *p* > 0.05, “*” for *p* ≤ 0.05, “**” for *p* ≤ 0.01, “***” for *p* ≤ 0.001, and “****” for *p* ≤ 0.0001.

## 3 Results

### 3.1 Alterations of Epigenetic Regulator Gene Aberrations in TCGA Data

**FIGURE 1:**
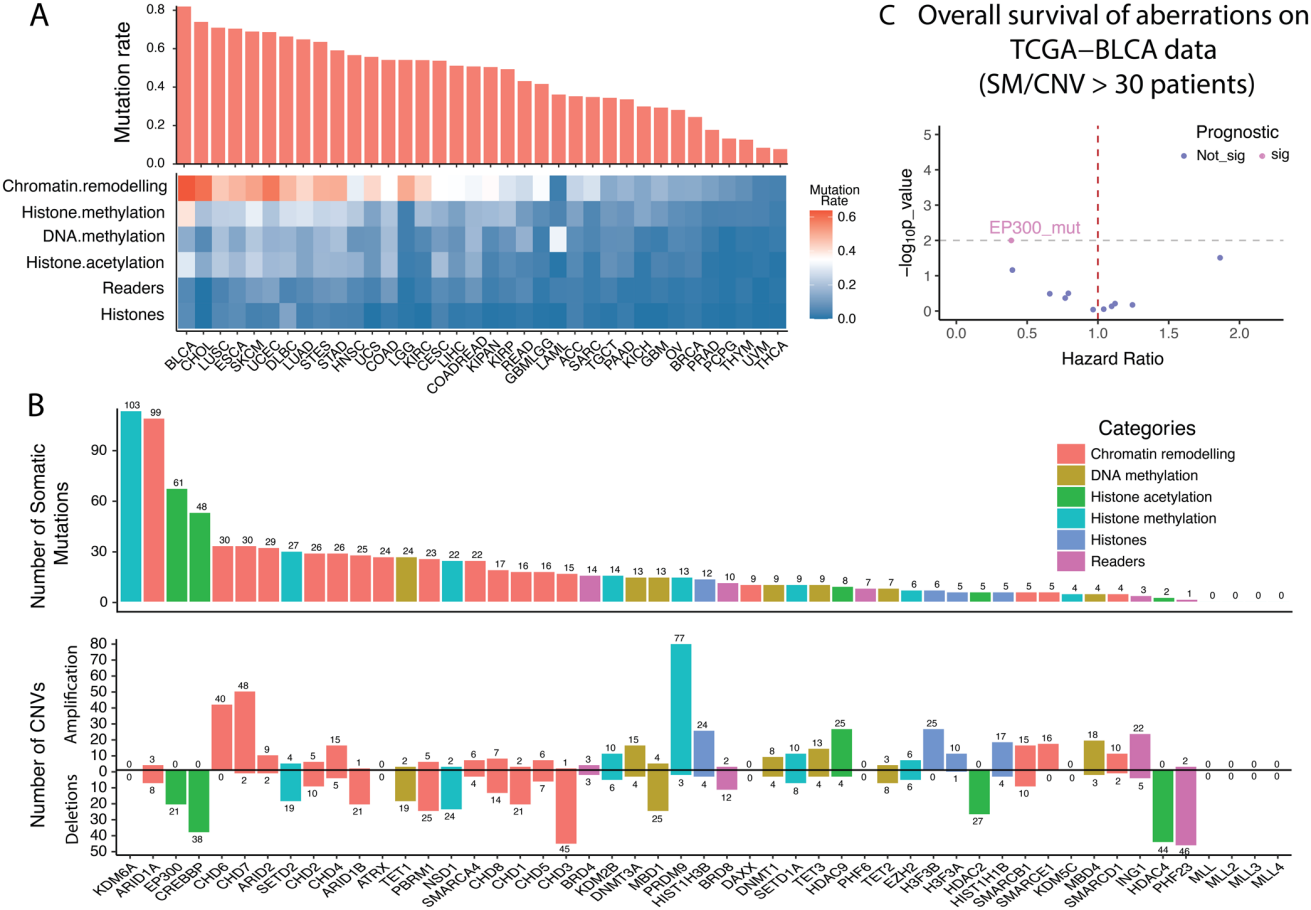
Predominance of SM (396 total samples) and CNV (indels, 408 total samples) in epiRG across the TCGA cancers (A) Panel of bar plot presents an overview of the somatic mutation frequencies of epiRG across all cancer types in TCGA. The heatmap panel highlights those genes involved in chromatin remodeling and histone methylation exhibit notably high mutation rates across several cancer types. This is followed by genes associated with DNA methylation, histone acetylation, reader proteins, and histones themselves. (B) Especially in BC: Quantitative Analysis of SM and CNV in all epiRG in BC samples. This figure comprises two panels representing the number of aberrations in each epiRG. Upper Panel: Displays the sample count of somatic mutations for each epiRG, with the number of mutations in samples plotted on the *Y*‐axis. Each bar represents a specific epiRG, which is identified along the *X*‐axis. The epiRG are categorized according to their role in epigenetic regulation, such as DNA methylation, chromatin remodeling, or histone modification. Lower Panel: Illustrates the CNV data, categorized as either amplifications or deletions. Amplifications are depicted by bars extending above the *Y*‐axis (from 0 upwards), whereas deletions are represented by bars extending below the *Y*‐axis (from 0 downwards). Like the upper panel, the *X*‐axis details each epiRG along with its corresponding category of epigenetic regulation. This bifurcated representation provides a comprehensive view of the genetic alterations in epiRG, illustrating the prevalence and types of SM and CNVs across the spectrum of epigenetic regulators. (C) Volcano plot to show the prognostic association of epiRG aberrations rations (SM/CNV > 30) to the overall survival (OS) using Cox proportional hazards model. *X*‐axis represents the hazard ratio (HR), and *Y*‐axis indicates log adjusted *p*‐values.

We analyzed the mutation landscape of epiRG across all TCGA cancers and found BC has the highest overall mutation rate (more than 80%) compared to the other cancer types. Quantitative analysis revealed chromatin remodeling genes exhibited the highest overall mutation rate in BC, followed by histone modification (Figure 1A). In BC samples, the most frequently mutated genes were *KDM6A* (103 mutations), *ARID1A* (99 mutations), *EP300* (61 mutations), and *CREBBP* (48 mutations) (Figure 1B: Upper panel). We also investigated the CNV changes of epiRG and found that, although several genes did not have high mutation rates, their CNV changed drastically in BC. For example, only 13 SM BC samples have *PRDM9* mutations, but *PRDM9* amplification was observed in 77, suggesting that they may be involved in different downstream mechanisms. Similarly, *HDAC4* and *PHF23* were barely mutated in BC, but deletions were observed in 44 and 46 of 408 samples, respectively (Figure 1B: Lower panel). Understanding these relationships is vital for developing targeted therapies tailored to individual genetic and epigenetic profiles. We binarized the status of epiRG's ARID1A‐mut, CHD6‐mut, CHD7‐mut, CREBBP‐mut, EP300‐mut, KDM6A‐mut, CHD6‐amp, CHD7‐amp, PRDM9‐amp, CHD3‐del, CREBBP‐del, HDAC4‐del, and PHF23‐del with the highest frequency rates (> 30 bladder samples) to perform survival analysis. The Cox model revealed that out of 13 epiRG, only EP300 exhibited a highly significant association with a *p*‐value of 0.01 and a Hazard Ratio (HR) of 0.39 (Figure 1C). These findings underscore the challenge of relying solely on epiRG aberrations for predicting prognosis, emphasizing the need for the development of alternative patient‐specific scores for aberrations.

### 3.2 Differentiation and Classification of Driver epiRG Aberration Signature Scores

**FIGURE 2:**
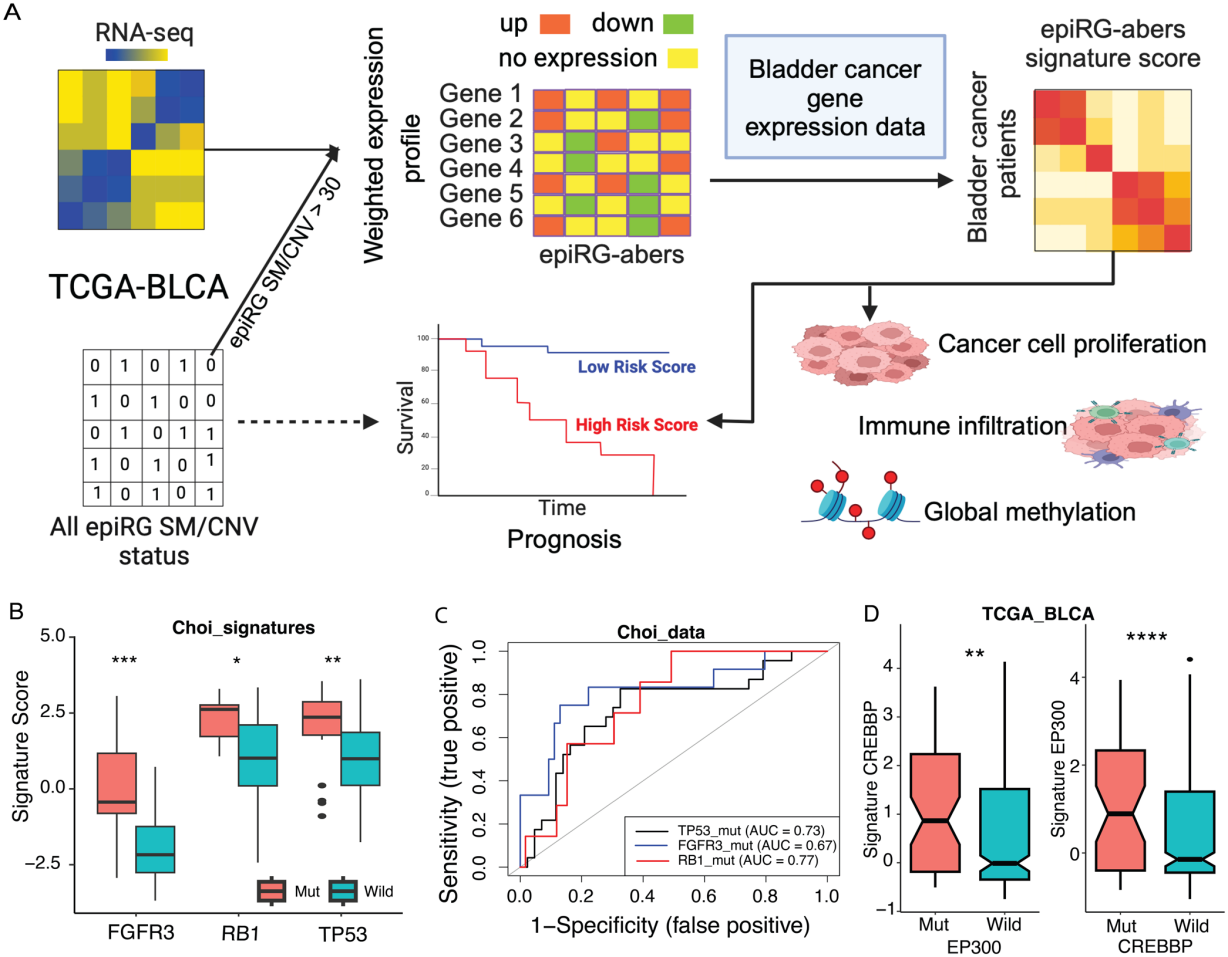
(A) Schematic representation illustrates the key components which includes the development of epiRG aberrations signature scores: A critical aspect of the diagram is the portrayal of how expression profiles of each epiRG aberrations derived from the integration of SM, CNV and transcriptomic data and, can be used to predict clinical outcomes. The diagram elucidates the process of signature scores development, encompassing the identification and correlation of these signatures with patient prognosis, interaction with the cancer cell proportion, immune cell infiltration and global DNA methylation changes. (B) This figure illustrates how the signature scores for *FGFR3*, *RB1*, and *TP53* genes can effectively differentiate Choi data with mutations from those with WT. (C) Receiver Operating Characteristic (ROC) curves evaluating the effectiveness of epiRG signature scores in distinguishing patients with TP53, FGFR3, and RB1 mutations from those with WT in a test dataset. (D) Cross‐prediction capability of signature scores in TCGA‐BLCA data: CREBBP‐mut signature scores demonstrates the ability to predict patients with EP300 mutations compared to WT patients and vice versa. The statistical significance was determined using Wilcoxon rank‐sum tests.

We defined the epiRG aberration signatures using TCGA‐BLCA data by integrating SM, CNV, and transcriptomic data (Figure 2A). To assess the effectiveness of these genes, we first evaluated their ability to distinguish between mutant and WT samples in the Choi bladder dataset. We observed that higher signature scores were significantly associated with samples harboring mutations in three genes (Figure 2B). This suggests a correlation between mutation frequency and signature scores, indicating potential underlying biological alterations similar to the effects of gene aberrations in tumor tissue. For the gene FGFR3, high signature scores are associated with patients carrying mutations, whereas lower scores are linked to WT samples. This relationship may vary across other genes due to biological interactions. Additionally, the predictive power of these signature scores was demonstrated by the area under the ROC curves (AUC) (Figure 2C), with classification efficacy quantified as AUCs of 0.77 for RB1, 0.67 for FGFR3, and 0.73 for TP53. We also observed improved prediction accuracy using signature scores derived from the TCGA‐BLCA data (Figure S1). These AUC values reflect each signature's ability to distinguish between mutated and WT samples, with higher AUC values indicating better predictive performance. Next, we sought to explore the intriguing possibility of cross predicting the gene signatures of CREBBP and EP300, which are widely expressed as transcriptional coactivators in BC [11]. Then, we assessed the co‐occurrence of mutations in the HAT complex genes in a cohort of 408 BC patients. We observed 62 patients with CREBBP mutations and 69 with EP300 mutations, with 13 patients exhibiting concurrent mutations in both genes (Table S1). This co‐occurrence may indicate that CREBBP and EP300 function within a shared regulatory pathway essential for maintaining normal cellular processes. Supporting this, we observed significantly higher signature scores for CREBBP‐mutated samples among patients with EP300 mutations, whereas lower scores were evident in patients with WT EP300 (Figure 2D). A similar pattern was observed for EP300‐mutated samples, which showed elevated signature scores in the presence of CREBBP mutations. From the Choi data and cross‐prediction analysis, it is evident that high signature scores are associated with samples harboring mutations. However, these scores are always dependent on their downstream pathways due to their aberration. These elevated signature scores suggest gene overexpression in patients, which can alter cellular functions and potentially activate oncogenic pathways within tumor cells. Next, we validated the effectiveness of our findings using independent BC datasets.

### 3.3 Association of Driver Gene Signatures With Prognosis Using Independent Datasets

Next, we investigated the prognostic association of driver aberration signatures using the GSE32894 dataset, which comprises 224 samples with survival information. Specifically, we generated signature scores for *TP53*, a known mutated gene in BC, which showed significant differentiation in the Choi testing dataset, supporting the use of these signatures for predicting patient survival.

**FIGURE 3:**
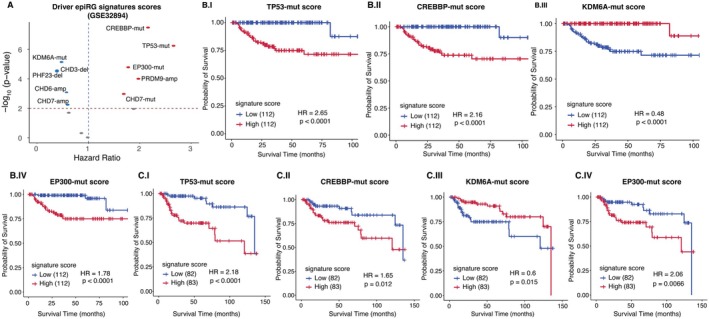
Evaluating the prognostic impact of driver epiRG aberration scores (*Z*‐scores) in BC using independent data sets GSE32894. (A) Volcano plots depicting the correlation between the hazard ratio (HR) and log adjusted *p*‐values for all signature scores derived from Cox proportional hazard models, providing a quantitative measure. Kaplan–Meier (KM) survival analysis in the context of driver TP53‐mut, CREBBP‐mut, KDM6A‐mut and EP300‐mut in BC patients in GSE32894 data (B: I, II, III and IV plots) and external validation GSE13507 dataset (C: I, II, III and IV plots).

In the univariate Cox proportional hazard analysis (Figure 3A), five signatures were significantly and negatively associated with prognosis (*p* < 0.01 and Hazard Ratios: HR > 1). This suggests that a higher burden of aberrations is linked to shorter survival, possibly by dysregulating key pathways in their presence. Conversely, five signatures showed a positive association with protection (*p* < 0.01 and HR < 1), indicating that lower signature scores correlate with poorer survival outcomes. These results clearly indicate that the development of cancer is driven by genetic and epigenetic aberrations that disrupt normal cellular regulation, highlighting the potential role of oncogenic pathway activation and its modulation through various mechanisms. For a better understanding, we focused on highly significant epiRG aberrations (*p* < 1.6e‐05) for stratification using the Log‐rank test (*p* ≤ 0.01, median as a cut off, Figure 3B,I to IV). Kaplan–Meier plots revealed that patients with high scores (red color) were associated with high risk and shorter overall survival time for TP53‐mut, CREBBP‐mut, and EP300‐mut. Conversely, high KDM6A‐mut scores (red color) had prolonged survival in all patients. Subsequently, we further validated the result in independent datasets (GSE13507) and observed similar results (Figure 3C,I to IV). Specifically, a high TP53 mutation signature score indicates defective pathway activity and confirms that our signature scores are strongly associated with patient prognosis [22]. Moreover, we integrated the mutation status of CREBBP and EP300, given their involvement in shared cellular processes [11, 46]. Interestingly, EP300CREBBP‐mut signature scores were significantly associated with poorer overall survival (HR > 1) in both datasets (Figure S2), suggesting that the overall co‐mutation burden of these genes may contribute to the same biological pathways.

**FIGURE 4:**
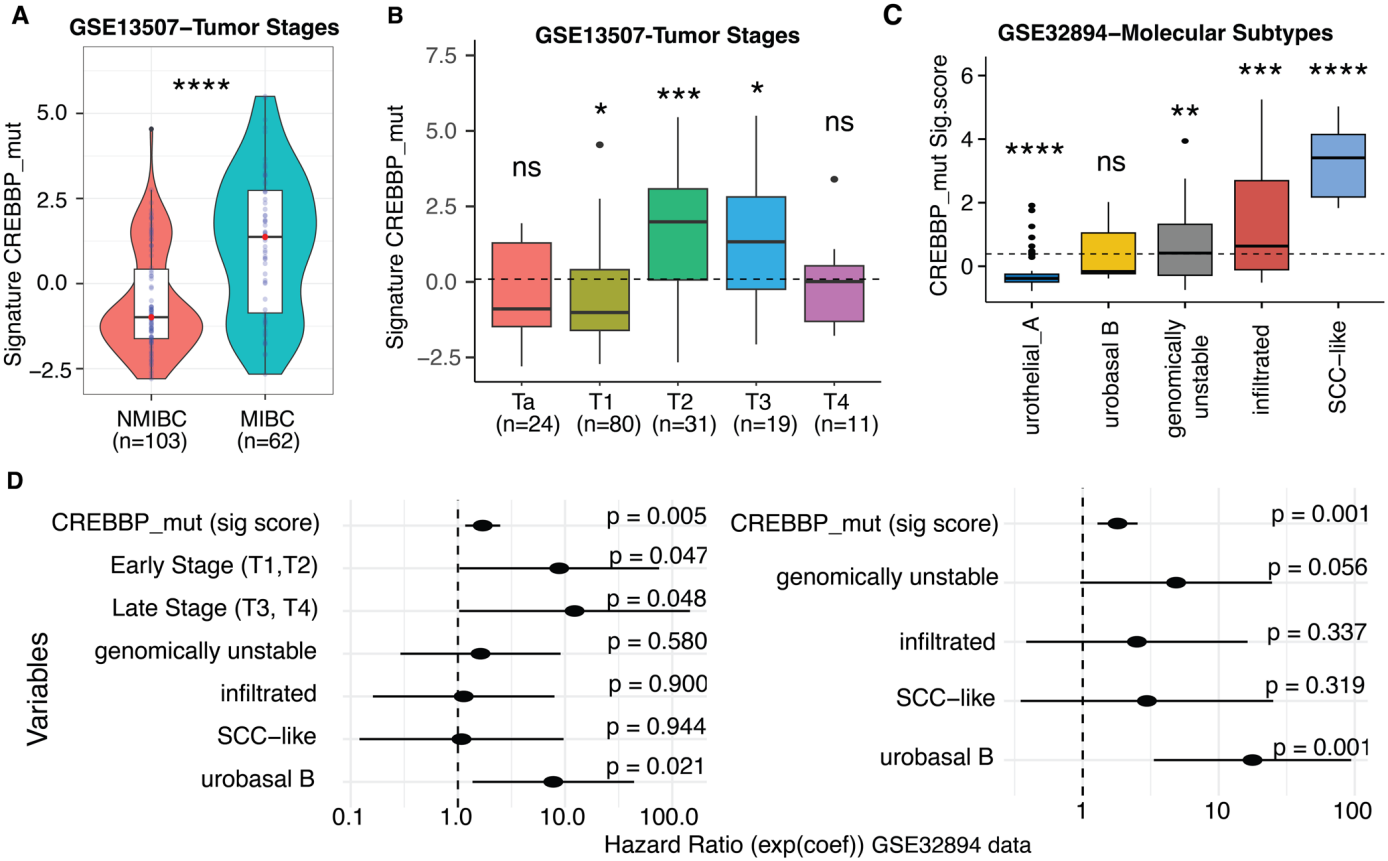
The CREBBP‐mut signature scores has the highest significance in prognosis for bladder samples: (A) Boxplot reveals the association of CREBBP‐mut with invasiveness (non‐ muscle invasive: NMIBC vs. muscle‐invasive: MIBC) and signature scores can significantly differentiate patients with MI and non‐MI. (B) Boxplots demonstrates that the signature scores of CREBBP‐mut can differentiate into different tumor stages in GSE13507 datasets (where non‐MI: Ta and T1, and MI:T2, T3 and T4 stages). (C) This plot presents the signature scores distribution of CREBBP mutation across different molecular subtypes in BC: Urothelial‐A, Urobasal B, Genomically Unstable, Infiltrated, and SCC‐like. This visualization helps compare the levels of CREBBP‐mut signature scores across these distinct subtypes, highlighting any significant variations or similarities in their distribution. (D) The forest plot serves to validate the prognostic value of CREBBP‐mut signature scores. In this analysis, the signature scores are evaluated within the framework of a multivariate Cox proportional hazards model. This model adjusts for various clinical factors, specifically tumor stages (Pre‐early‐Ta samples, Early stage‐T1 & T2 and Late stages‐T3 & T4) and molecular subtypes.

We further investigated the activity of highly significant driver CREBBP‐mut scores across different clinical groups (Figure 4). Specifically, we examined invasive BC patients, with the majority of tumor cases across both early and late clinical stages [24]. Notably, high scores were significantly associated with muscle‐invasive BC (MIBC), compared to non‐muscle invasive BC (NMIBC) (*p* = 5.3E‐07, Figure 4A) in the GSE13507 dataset. To confirm the association of signature scores with tumor stages (Ta, T1, T2, T3, and T4), we observed a notable distribution of mean scores across T‐stages (Figure 4B), with an increasing trend from early to advanced stages (*p* = 3.5E‐08). Additionally, T1, T2, and T3 stages exhibited statistically significant differences (*p* < 0.05). Cox univariate analysis further revealed a significant prediction of clinical outcomes in patients with CREBBP‐mut scores, as well as factors, such as age, invasiveness, and tumor stage (Table S2). These findings are consistent with trends observed in the GSE32894 dataset, where higher scores were associated with more advanced tumor stages, reflecting high mutation rates (Figure S3A).

To understand the association between signature scores and other clinical outcomes, we analyzed molecular taxonomy profiles of BC and tumor stages [41] in the GSE32894 dataset. Urothelial‐A patients had low scores associated with better survival, whereas SCC‐like patients had high scores linked to poorer survival outcomes (*p* = 2.2E‐16) (Figures 4C and S3B). Notably, patients with elevated scores are associated with the worst prognosis in multivariate Cox regression analysis, which includes tumor stages and molecular subtypes (Figure 4D). Specifically, the Urobasal B subtype, which often demonstrates aggressive clinical behavior and poorer prognosis, showed higher scores along with advanced stages (Table S3). The Urobasal B subtype had a relatively higher Hazard Ratio (HR), indicating a worse prognosis, followed by the genomically unstable, SCC‐like, and infiltrated subtypes. Intermediate scores in the genomically unstable subtype suggest varying prognostic implications, highlighting the cellular proliferation dynamics captured by these scores. Higher scores in the infiltrated subtype correlated with increased cell infiltration, as reflected in the CREBBP‐mut. Taken together, these results demonstrate that CREBBP‐mut scores are strongly correlated with clinical factors, such as invasiveness, tumor stages, and molecular subtypes.

### 3.4 Epigenetic Regulator Gene Aberrations Are Linked With Cell Proliferation and Infiltration Changes in the TME

**FIGURE 5:**
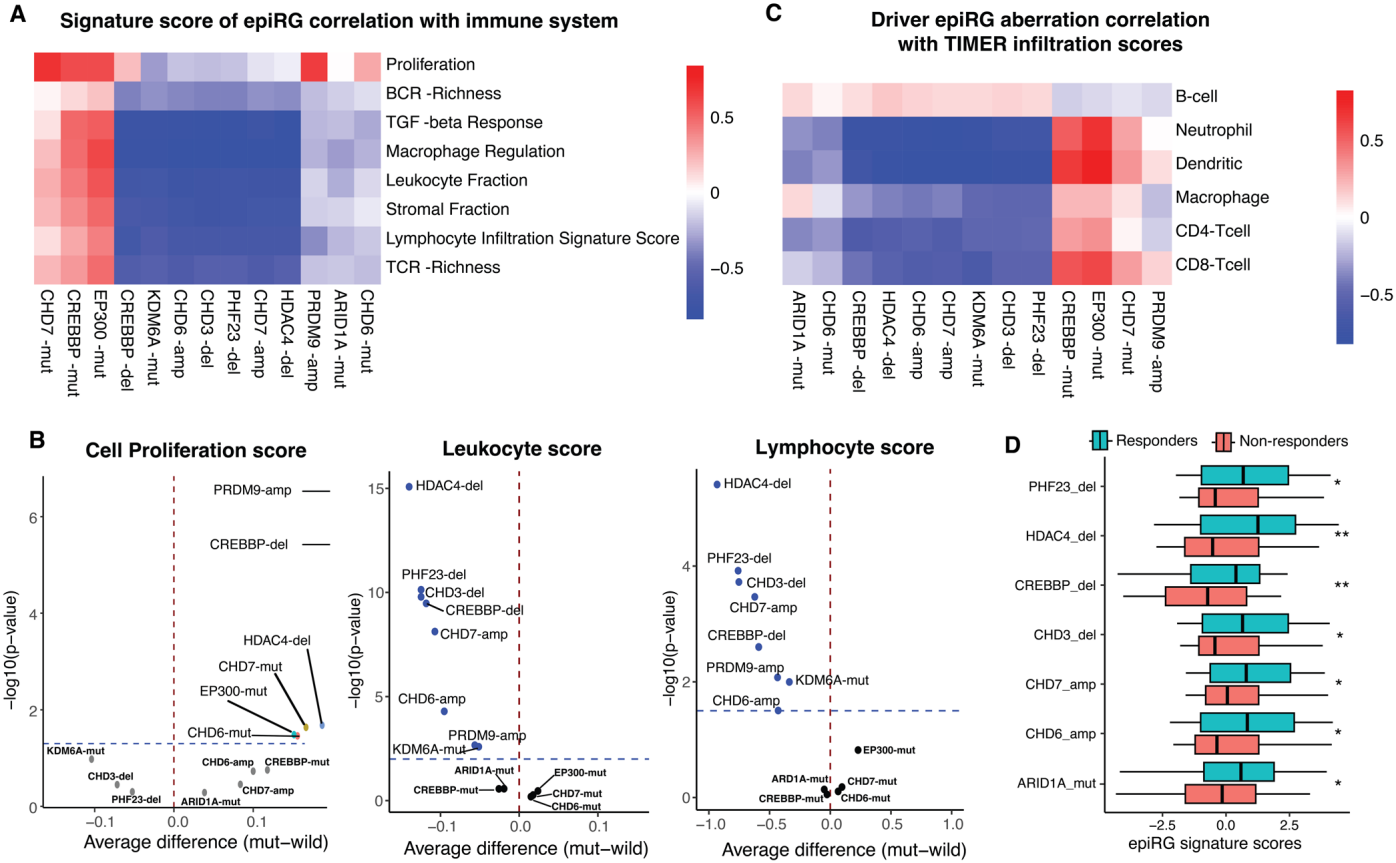
Associations of epiRG aberration scores with cell proliferation and immune infiltration: (A) Correlation of epiRG aberration signature scores to the Immune scores. The intensity of red color represents a strong positive correlation, whereas blue color indicates a negative correlation in both Figure A and C. (B) Volcano plots visualize the correlation between epiRG aberrations and the immune microenvironment, using Thorsson's classifications, *X*‐axis represents the difference between epiRG aberrations to WT, *Y*‐axis represents the statistical significance calculated using paired *t*‐test. (C) A heatmap of spearman correlation coefficients capture the relationship between specific epiRG aberrations to the six immune cell types of TIL's scores from TIMER data. (D) Using epiRG aberrations signature scores, this box plot represents the comparison of urothelial BC patients who responded to immune checkpoint blockade treatment with those who did not. In this comparison, the Wilcoxon rank sum test was employed to determine statistical significance between the two groups (*p*‐values). This approach helps in identifying whether there is a significant difference in the epiRG aberrations signature scores between patients who benefited from the treatment (responders) and those who did not (non‐responders).

We analyzed the relationship between cancer cell aberrations and the immune system. Some signatures were positively correlated with proliferation, whereas most showed a negative immune association (Figure 5A), suggesting aberrations shape the TME to promote BC progression. High expression scores in CHD7‐mut, CREBBP‐mut, EP300‐mut, PRDM9‐amp, CREBBP‐del, and CHD6‐mut patients correlated with increased proliferation, whereas immune‐suppressive signatures (e.g., KDM6A‐mut) linked to lower proliferation. This implies some aberrations drive poor prognosis via proliferation, whereas others suppress it, increasing immune infiltration and improving outcomes. Aberration‐positive patients had higher proliferation but reduced lymphocyte/leukocyte infiltration (Figure 5B), confirming genomic aberrations influence gene expression.

Using Tumor Immune Estimation Resource (TIMER) data [47], we found patients with *CREBBP*, *EP300*, *CHD7* mutations and PRDM9 amplifications associated with low B‐cell infiltration, potentially aiding tumor growth. Conversely, other mutations correlated with higher B‐cell infiltration and worse prognosis, suggesting immune evasion or surveillance roles (Figure 5C, Table S4). Notably, patients with *CREBBP*, *EP300*, *CHD7* mutations increased CD8+, CD4+ T cells, macrophages, neutrophils, and dendritic cells, whereas PRDM9 amplification weakly correlated with CD8+ T cells and dendritic cells. Greater immune infiltration suppressed proliferation and improved prognosis. These findings were supported by immune pathway analysis, revealing aberrant signatures interact with 12 key immune pathways (Figure S4, https://github.com/venu887/Epi_Bladder‐cancer/blob/main/Input_files/Suppl_immpath.csv). For example, the “intestinal immune network for IgA production”, “Angioimmunoblastic lymphoma down”, and “Bohn primary immunodeficiency syndrome up” pathways are related to interactions with B‐cells. We also observed high correlation coefficients (|*ρ*| ≥ 0.7) between immune cell marker genes and these signatures, highlighting interactions with other genes that play critical roles in BC (Figure S5, https://github.com/venu887/Epi_Bladder‐cancer/blob/main/Input_files/Suppl_immgene.csv). Moreover, we identified key genes within the FGFR family that may play an important role in immune interactions and tumor progression in BC.

To assess clinical relevance, we examined how identified aberrations influence PD‐L1 immunotherapy response. High signature scores correlated significantly with positive PD‐L1 treatment outcomes (Figure 5E). Specific genetic alterations, such as ARID1A‐mut, CHD6‐amp, CHD7‐amp, CHD3‐del, CREBBP‐del, HDAC4‐del, and PHF23‐del were significantly associated with treatment responders, suggesting that aberrations linked to increased immune infiltration may enhance immunotherapy efficacy. Conversely, ARID1A‐mut absence correlated with aggressive progression, metastasis, and impaired checkpoint signaling [48, 49]. These findings highlight the potential of targeting epiRG aberrations and immune pathways to optimize immunotherapy. Genetic signatures influencing immune infiltration could guide personalized treatment strategies, improving prognosis and therapeutic response.

### 3.5 Association of epiRG Aberrations With a Global DNA‐Methylation Change

**FIGURE 6:**
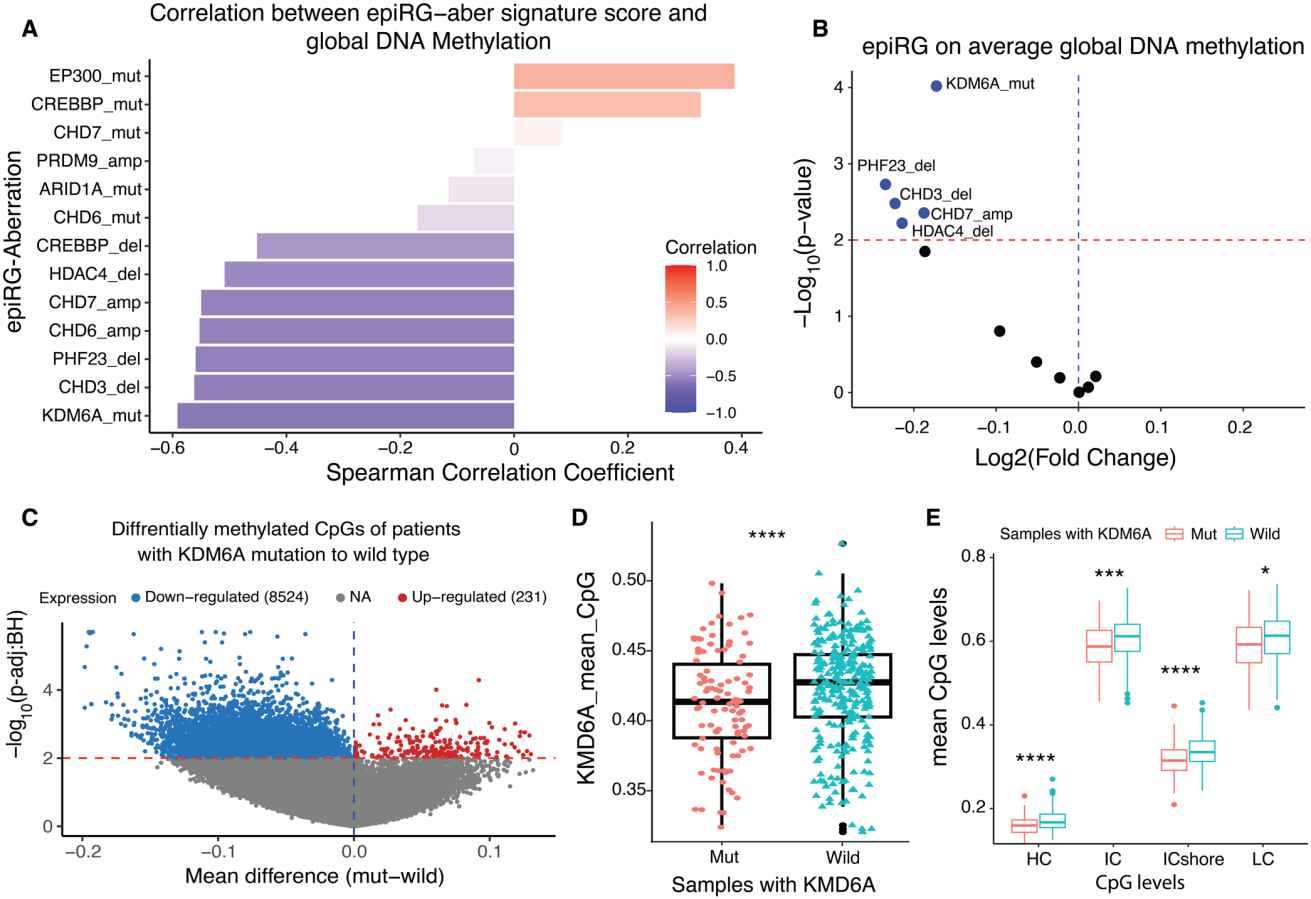
Effect of epiRG aberrations on DNA methylation changes: (A) Correlation analysis with global methylation with signature scores, where red color intensity indicates positive correlation and blue color intensity indicates negative correlation. (B) Volcano plot displays global DNA methylation changes in patients with aberrations in epiG's: Assessed median methylation levels through paired *t*‐tests and calculated fold changes using average methylation values as Log 2‐fold changes. Statistically significant *p*‐values were plotted on the *y*‐axis against magnitude of change on the *x*‐axis. (C) Differential methylation analysis of patients with KDM6A‐mut to the WT. Blue colored dots indicate downregulated CpGs and red colored dots indicates upregulated CpGs. The *X*‐axis is the mean differences of two groups (Mutation—WT) (D) This box plot illustrates the average methylation levels in patients who have mutations in the KDM6A gene (KDM6A‐mut), and the *Y*‐axis contains log adjusted *p*‐value. (E) This box plot shows the methylation distribution of KDM6A mutations across four different categories of CpG sites.

Epigenetic regulator genes directly interact with DNA methylation in cancer due to aberration reprogramming. In our final analysis, we sought to validate DNA methylation changes due to epiRG aberrations. We observed a strong negative association between KDM6A‐mut signature score and other aberrations associated with bladder patients with PD‐L1 responders, and a strong positive correlation with CREBBP‐mut and EP300‐mut scores (Figure 6A). We confirmed a significant positive association between patients with KDM6A‐mut, PHF23‐del, CHD3‐del, CHD7‐amp, and HDAC4‐del status on average DNA methylation levels (Figure 6B). A global differential methylation analysis comparing aberrant samples to WT patients revealed the number of upregulated and downregulated CpG levels across all driver epiRGs. This analysis demonstrated a high deviation of 94.72% in differential methylation of CpGs when comparing *KDM6A* mutant patients to WT patients (Table S5). This was followed by deviations of 91.60%, 88.89%, and 88.06% for *CREBBP*, *ARID1A*, and *EP300* patients, respectively. Notably, when compared to differentially methylated CpGs, the percentage of the highest deviation was observed in the KDM6A‐mutant samples (Figure 6C). Prior research has revealed that the loss of KDM6A‐mut activates FGFR3, leading to reduced expression of luminal genes and promoting a more basal, de‐differentiated cellular state in BC [50]. Additionally, the loss of KDM6A‐mut creates an epigenetic state that drives tumor growth and is crucial for pathogenesis and treatment [16, 51].

Moreover, Figure 6D revealed that KDM6A mean CpG levels significantly lower methylation levels in samples with mutations compared to WT samples (*p* = 9.5e‐05), indicating that KDM6A mutation corresponds to low CpG methylation. Our analysis further explored the differences in CpG classes between KDM6A mutation and WT, examining the methylation levels in four groups. The number of CpG probes in each group was as follows: 153,859 in HC, 118,727 in IC, 33,955 in IC‐shore, and 178,971 in LC. We observed that the density of these four CpG levels was highly associated with hypomethylation in patients with mutations: IC‐shore (*p* = 7.5e‐06), HC (*p* = 1.7e‐05), IC (*p* = 0.0005), and LC (*p* = 0.016) (Figure 6E). Bladder samples harboring KDM6A mutations exhibit a tendency towards hypomethylation. Low signature scores associated with KDM6A‐mut were observed, indicating potentially diminished gene expression or altered genomic features related to KDM6A. These low scores are negatively correlated with global DNA methylation levels leading to potential impact on the overall methylation status of CpG sites, suggesting a propensity towards hypomethylation of CpG sites associated with KDM6A mutations. Such hypomethylation events can have wide‐ranging consequences on gene regulation and cellular processes. Studies suggest that KDM6A mutations can influence the activity of B‐cells [52, 53]. This hypomethylation is further linked to immune infiltration dynamics, indicating a positive association with infiltration of B‐cells in conform involvement in BC adaptive immune response. Importantly, these findings have clinical implications as low signature scores associated with KDM6A mutations are linked to poor survival.

A detailed overview of the 13 epiRG signature scores and their association with clinical and immunological features can be found in Table S6. This table summarizes key observations across multiple datasets, including prognostic relevance, immune cell correlations, and prediction of patient response with immunotherapy.

## 4 Discussion

In this study, we define gene signatures for the epiRG aberrations, which are frequently observed in BC patients [6, 13]. Although patient stratification based on SM/CNV has been explored, its prognostic value remains limited when used alone. Therefore, developing novel signature scores that capture the functional impact of these aberrations is essential for enhancing therapeutic strategies and improving the prediction of clinical outcomes in BC [12, 25]. This study provides evidence that genetic changes in epiRG are not dominated by a single mechanism but are influenced by interactions with other genes and contributions from multiple pathways. We tested the signature scores using independent datasets containing patients with mutation status. Specifically, the Choi dataset confirmed that patients with mutations in *FGFR3*, *TP53*, and *RB1* have high scores. *TP53* is a well‐studied oncogene, and its mutation is linked to poor survival and disease progression in BC [39, 54]. Our TP53‐mut signature scores were higher in mutated cases in the Choi data and associated with worse survival later applied on GEO datasets. These scores effectively distinguished TP53‐mutant from wild‐type patients, showing strong predictive performance (high AUC) in both the Choi dataset and TCGA‐BLCA dataset. FGFR3 mutations are recognized as oncogenic drivers and promising therapeutic targets in BC [55], whereas RB1 mutations alone may not reliably predict prognosis but can still influence disease progression [56]. Using signature scores, we can improve prognostic prediction in patients with aberrations.

Further, we demonstrated the effectiveness of epiRG signature scores for predicting prognosis in BC. A positive association with KDM6A‐mut (HR < 1) signature scores and a negative association with CREBBP‐mut, EP300‐mut, TP53‐mut (as an external evaluation) (HR > 1) was highly significant (*p*‐value < 0.01) in GSE32894 data. Also, a highly significant prognosis was observed in the HAT complex (EP300CREBBP‐mut, HR > 1). We showed that high scores of CREBBP‐mut, EP300‐mut, TP53‐mut, and EP300CREBBP‐mut tend to have a poorer prognosis than those with low scores. Jason et al. [11] suggested that higher mutations in *EP300* and *CREBBP* are harmful and can control transcriptional profiles, whereas lower mutations are more likely benign and correlated with invasiveness. Our scores for CREBBP‐mut and EP300‐mut explain that these are closely related to the same biological processes [57]. Additionally, high EP300CREBBP‐mut scores are associated with patients harboring the mutations. KM‐plot indicates that high scores are linked to a poor survival rate. This represents that mutated patients share common downstream signaling pathways. Targeting this HAT complex is currently under preclinical investigation to treat BC due to its involvement in progression [11, 58, 59]. Investigating targeted therapies against this complex represents an important avenue for future research. However, elucidating their transcriptional coactivator mechanisms will require a separate, comprehensive study beyond the scope of the present work.

Furthermore, our study highlights the clinical relevance of highly significant CREBBP mutation signature scores in BC. Although previous work by Xu et al. [60] failed to establish CREBBP mutation status alone as a significant predictor of OS, even within treatment cohorts, our comprehensive signature scores approach successfully demonstrated its prognostic value. We found that high CREBBP‐mut scores were significantly associated with adverse clinical outcomes, including advanced pathological stages and aggressive molecular subtypes.

These findings align with established mechanisms of bladder tumorigenesis, where Chen et al. (2022) demonstrated that CREBBP mutations disrupt histone acetylation patterns, leading to transcriptional dysregulation in BC [61]. Gongmin et al. (2020) further showed that EP300 mutations drive oncogenic progression through impaired chromatin remodeling [15], with inactivation of both CREBBP and EP300 dysregulating key pathways [59]. Our results linked these epiRG alterations to immune modulation during progression, where high signature scores correlated with tumor proliferation and concurrent immune activation via upregulated immune genes/pathways. In BC patients, CREBBP mutations were associated with immunotherapy responses [46], characterized by reduced lymphocyte/leukocyte infiltration. Notably, IL6 was identified as a promoter of MIBC progression due to its elevation in CREBBP mutated patients, enhancing immune suppression [47]. This aligns with our broader analysis implicating chemokine ligands (CCL family), immune checkpoints (PD‐L1/CD274, PDCD1LG2, HAVCR2), and cell surface markers in shaping tumor immune phenotypes.

Similarly, CHD7‐mut signature scores demonstrated a comparable positive association with immune activity, where lower scores correlated with reduced immune cell infiltration in mutation‐positive patients. This pattern mirrors the immune‐modulatory effects observed with CREBBP/EP300 alterations, further supporting the role of epiRG dysregulation in shaping the TME. Immune infiltration is a dynamic process with immune cells interacting within the TME. Higher expression scores in patients with some aberrations lead to high cell proliferation and increased infiltration of leukocytes and lymphocytes, whereas others show the opposite action in the TME. We correlated epiRG scores with six major immune cell types and observed certain epiRG scores (CHD7‐mut, CREBBP‐mut, EP300‐mut) positively correlated with tumor proliferation. Thus, tumor samples with these aberrations had higher infiltration levels, especially CD8^+^ and CD4^+^ T cells. In contrast, other aberrations exhibited very low or negative correlation with tumor proliferation, which were associated with reduced immune cell infiltration. Notably, patients with low epiRG scores often carrying these mutations had poor OS (Example: KDM6A mutation). This negative correlation likely reflects an immune‐excluded TME characterized by minimal or absent immune cell infiltration. For example, Bcells releaseantibodies to fight against tumor cells and influence the reduction of cell proliferation. This is confirmed by interactions with immune genes like CD270 and B‐cell immune pathways.

Next, high scores of KDM6A‐mut are associated with good prognosis; *KDM6A* mutation decreases *KDM6A* gene expression [16]. Inactivation of this mutation acts as a tumor suppressor and reduces B‐cell activation in BC [62]. Notably, our integrated scoring model outperformed individual mutation status in prognostic stratification, highlighting its clinical relevance. Consistent with findings from Chen et al. [53], who reported that KDM6A mutations suppress antitumor immunity by negatively regulating immune signaling pathways, our results demonstrated that high KDM6A‐mut signature scores correlate with reduced immune cell infiltration.

Consistent with findings from Chen et al. [53], KDM6A mutations were associated with significantly reduced infiltration of key immune populations, including macrophages, CD8+ T cells, neutrophils, and dendritic cells. Our signature scores were consistent with these observations, showing strong negative correlations with CD8+ T cells, CD4+ T cells, macrophages, dendritic cells, and neutrophils, but unexpectedly revealed a slight positive association with B‐cell infiltration. Further aligning with Chen et al. [53] our analysis demonstrated that KDM6A‐mut signature scores correlate with downregulation of critical immune pathways, particularly TCR, BCR, and chemokine signaling, highlighting a conserved mechanism of immune suppression in BC. Notably, Kobatake et al. [63] identified that KDM6A mutations upregulate pro‐tumorigenic genes (CCL2, IL6, CXCL1) via the chemokine signaling pathway, driving M2 macrophage polarization and STAT3 activation. Our results complement these findings, as low KDM6A‐mut signature scores associated with favorable prognosis showed a significant negative correlation with these pathogenic factors. Together, these findings demonstrate how loss of KDM6A mutations creates an immunologically cold tumor microenvironment through both cellular depletion and pathway suppression. This effect appears mediated through negative interactions with both immune‐related genes and pathways, further compromising antitumor immune responses.

In addition, we also evaluated epiRG scores in relation to global DNA methylation levels and found KDM6A‐mut scores were highly correlated. Results indicated that KDM6A mutated samples are associated with lower methylation levels compared to WT samples. A study on BC patients revealed that higher levels of immune infiltration were associated with significant methylation changes [64], implying that lower infiltration correlates with reduced CpG methylation. Furthermore, we identified that KDM6A‐mut patients have low CpG levels, which may contribute to better prognosis.

A key observation using Thorsson [33] is that immune expression scores were correlated with epiRG signature scores in BC patients. Further, these expression scores revealed that patients with HDAC4 deletion tend to have higher tumor proliferation compared to WT. This resulted in fewer lymphocyte and leukocyte cells in HDAC4 deletion patients. This inverse relationship between HDAC4 deletion status and immune cell recruitment suggests that HDAC4 may function as an immune checkpoint regulator in BC pathogenesis [65]. Our results support and confirm that high HDAC4‐del signature scores predicted the patients undergoing PD‐L1 immunotherapy responders. Both expression scores and our signature scores consist of the same results; additionally, our scores predicted responders. Correlation with TIMER indicates that B‐cells play a vital role in these epiRG aberrations, with observed infiltration leading to reduced cell proliferation. This finding was supported by regulation with specific immune genes, displaying varied responses based on the type of epiRG aberrations. Crucially, gene panels have identified prognostic biomarkers with significant values in immunotherapy. Our study further explored the impact of epiRG aberrations scores on patient response to PD‐L1 immunotherapy. We found that certain epiRG aberrations significantly correlate with immunotherapy responses, suggesting their potential as biomarkers for optimizing treatment strategies. This research highlights the significant role of epiRG aberrations scores in modulating immune responses in BC, offering insights for more personalized treatment approaches.

In summary, our comprehensive study utilized 13 epiRG aberration signature scores derived from an integrative analysis using TCGA‐BLCA data. We validated their associations with prognosis and confirmed their roles in cell proliferation and immunological interactions in TME. Additionally, our correlation analysis highlighted the potential of these signature scores to provide insights into tumor dynamics and immune responses. These findings can aid future clinical applications, as these signatures could enhance prognostic accuracy and guide personalized precision medicine strategies for immunotherapy. Further research is warranted to explore the therapeutic implications of targeting these epigenetic alterations in BC. A limitation of our study is that we did not focus on specific mutation types, as this falls beyond the scope of our current objectives. However, investigating the role of mutation types in relation to epiRG remains an important direction for our future research.

## Author Contributions

**Venugopalareddy Mekala:** resources, formal analysis, data curation, methodology, validation, visualization, writing – review and editing, writing – original draft, investigation, conceptualization. **Yupei Lin:** writing – review and editing, supervision. **Xiang Wang:** writing – review and editing, supervision. **Naail Chowdhury:** writing – review and editing. **Jianrong Li:** writing – review and editing, supervision. **Chao Cheng:** conceptualization, investigation, methodology, writing – review and editing, project administration, supervision, data curation, funding acquisition, formal analysis, resources, writing – original draft, software, validation, visualization.

## Ethics Statement

The authors have nothing to report.

## Conflicts of Interest

The authors declare no conflicts of interest.

## Supporting information


Appendix S1.

## Data Availability

All datasets used in this study were obtained from previously published and publicly available sources. The Cancer Genome Atlas (TCGA) cancer patient data was accessed via FireBrowse (http://firebrowse.org), and Gene Expression Omnibus (GEO) datasets are available under accession numbers GSE48075, GSE32894, and GSE13507. Immune landscape expression data and associated results were obtained from the supp lemental tables of NIHMS958212 (Table S2) [33]. The preliminary data used for this analysis can be freely accessed and reproduced via the following link: https://github.com/venu887/Epi_Bladder‐cancer/blob/main/BLCA_Priliminary_data.txt. The code used to generate the results and figures presented in this study is available at: https://github.com/venu887/Epi_Bladder‐cancer.
