# Supplementary material for: Integrative Analysis Reveals the Prognostic Effects of Epigenetic Regulators in Bladder Cancer
Source: Cancer Med. 2025 Jul 22;14(14):e71057. doi: 10.1002/cam4.71057 (PMC12281021; doi:10.1002/cam4.71057)
Supplement: Supplementary file 1 — Appendix S1. [file CAM4-14-e71057-s001.zip › cam471057-sup-0001-AppendixS1.docx]

**Integrative analysis reveals the prognostic effects of epigenetic regulators in ­bladder cancer**

­­­Venugopalareddy Mekala ^1,2,3^, Yupei Lin^1,2,3^, Xiang Wang^1,2,3^, Naail Chowdhury^1,2,3^, Jianrong Li^1,2,3^, Chao Cheng^1,2,3,^ *

^1^Department of Medicine, Baylor College of Medicine, Houston, TX 77030, USA

^2^Dan L. Duncan Comprehensive Cancer Center, Baylor College of Medicine, Houston, TX 77030, USA

^3^The Institute for Clinical and Translational Research, Baylor College of Medicine, Houston, TX 77030, USA

∗Corresponding author [chao.cheng@bcm.edu](mailto:chao.cheng@bcm.edu)

**Supplementary Methods:**

**Curation and processing the data for cell proliferation and TME:**

To unravel the key role of epiRG-aber in cell proliferation and TME within this context is pivotal for insights into immunotherapy outcomes. Our primary dataset encompassed cancer immune scores characterized by immunogenomics methods across 33 TCGA cancers (1). Curated extensive data and validated (Throsson et al., Supplementary Table ST1), including contributions from other studies, providing a rich resource to explore the role of epiRG-aber. We extracted bladder cancer information related to cell proliferation and tumor immune microenvironment (TIME), then performed Spearman's rank correlation coefficient (SCC) to understand the strength and association. We also performed significant differentiation of epiRG-aber with wild type samples using signatures scores. Secondly, we have downloaded tumor-infiltrating lymphocytes score (TIL-score) from TIMER2.0 (2) for six immune cell types (B cells, CD4 T-cell, CD8 T-cell, Neutrophil, Macrophage, and Dendritic cell). Then, performed correlation between TIL-score and epiRG-aber scores to understand the role and infiltration of immune cells on tumor surrounded by TME (3). Thirdly, we performed a correlation analysis with immune signature gene sets associated with immune pathways from BIOCARTA, KEGG, PID and REACTOME datasets from Molecular Signature Database (MsigDB) curated C2 gene sets (4). For this, Gene Set Variation Analysis (GSVA) was implemented through the R-package *“gsva”*, was utilized to estimate the variation of pathway activity across a sample population (5). This involved using normalized RNA-seq values and selecting genes with expression values in at least 90% of bladder samples. We selected minimum of 20 and maximum of 500 as a cutoff on the size of a gene set after gene identifies while using GSVA. Computed SCC between epiRG-aber signature scores to enrichment scores creating a downstream pathway inside the tumor cells due to changes in epiRG. Fourthly, we retrieved 1359 (6-8) marker immune gene lists and corresponding expression data were isolated from TCGA-BLCA RNA-seq. Performed SCC with normalized immune gene expression with epiRG-aber signature scores (Same as above immune pathways) (9, 10) to understand the downstream immune gene effect on pathways in tumor. Finally, we retrieved RNA-seq and clinical information from urothelial bladder cancer patients who underwent PD-L1 immunotherapy treatment response and non-response also raw data. The human raw gene expression data is available at the European Genome-phenome archive (EGA) under accession numbers EGAF00005211598 (11). This analysis targeted patients receiving PD-L1 immunotherapy and signature scores for epiRG-aber were computed using RNA sequencing data from urothelial bladder cancer samples by employing the BASE algorithm. Significance threshold was established at a p-value of < 0.05 (Wilcoxon rank sum test).

**Procession of DNA-Methylation data for analysis:**

To enhance our comprehension of epiRG alterations, we investigated their impact on DNA methylation in TCGA-BLCA samples. At first, we performed SCC analysis to understand the impact of epiRG-aber score in DNA methylation of epiRG’s. Our study utilized a curated dataset of matched samples only. Further, for each sample each with CpG beta values ranging from 0 (Hypo methylation) to 1 (Hyper methylation) were selected. These samples were categorized into two groups: epiRG status. We calculated global methylation levels using all epiRG-aber status using all CpG’s. Then, performed differential methylation levels of each epiRG-aber. Further, recognizing that CpG dinucleotides are not randomly distributed throughout the genome, we considered CpG Islands, regions highly enriched with CpG’s and often associated with gene promoter regions. These regions were classified as high-density CpG island (HC), intermediate-density CpG island (IC), intermediate-density CpG island shore (IC-shore), and non-island (LC) (12). In our analysis, we assessed how epiRG alterations differed among these four groups. To identify global methylation changes, we conducted statistical t-tests between epiRG-aber to wild-type group samples, calculating q-values using the “BH” method for multiple testing correction. CpG sites were then categorized as upregulated or downregulated based on their t-scores and q-values. Specifically, CpGs with t-scores > 0 and q-values < 0.01 were considered upregulated, while those with t-scores < 0 and q-values < 0.01 were deemed downregulated CpG’s for each epiRG-aber.


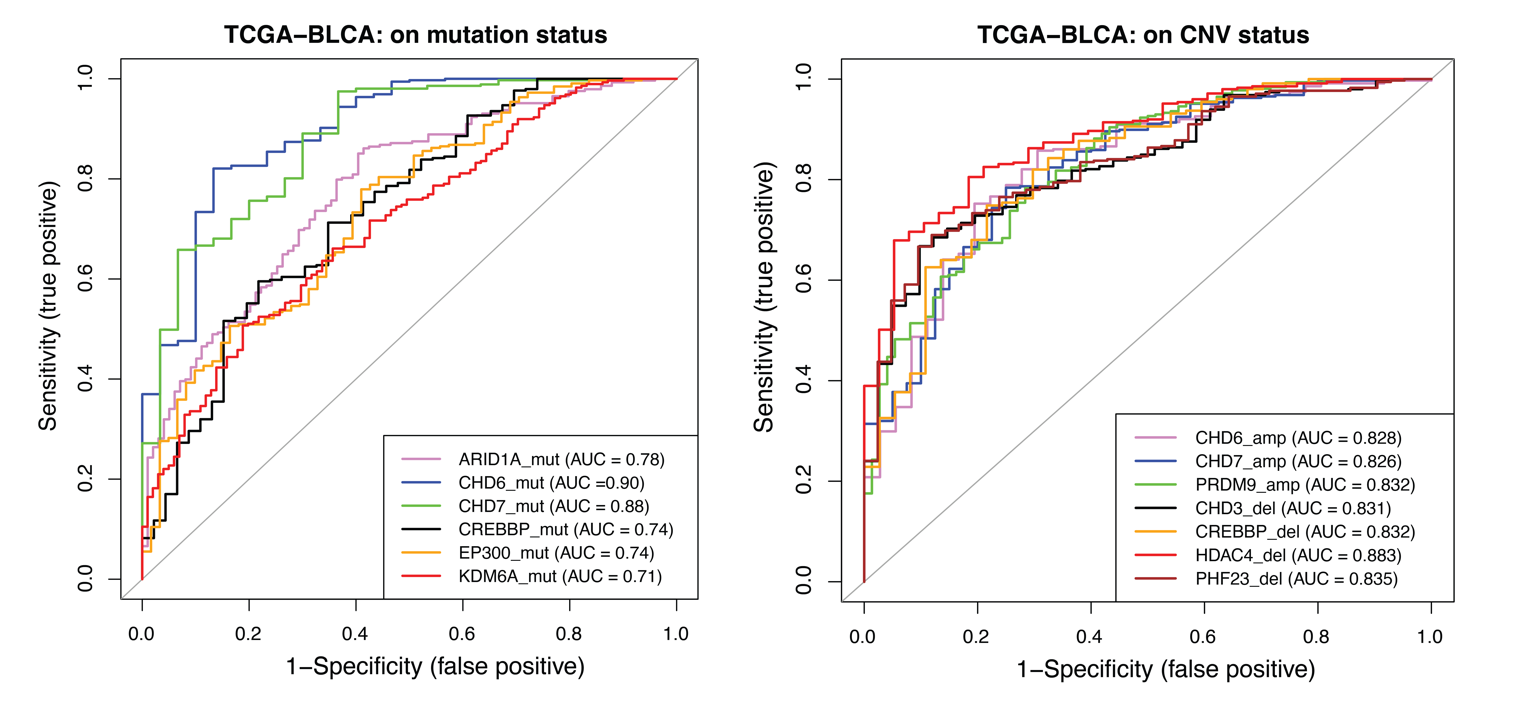


**Figure SF1:** ROC curves and the AUC of aberrations (SM/CNV ≥ 30 patients) epiG signature scores on patient aberration status prediction. We calculated signature scores using TCGA-BLCA data, by using gene signatures that are derived from TCGA-BLCA data.


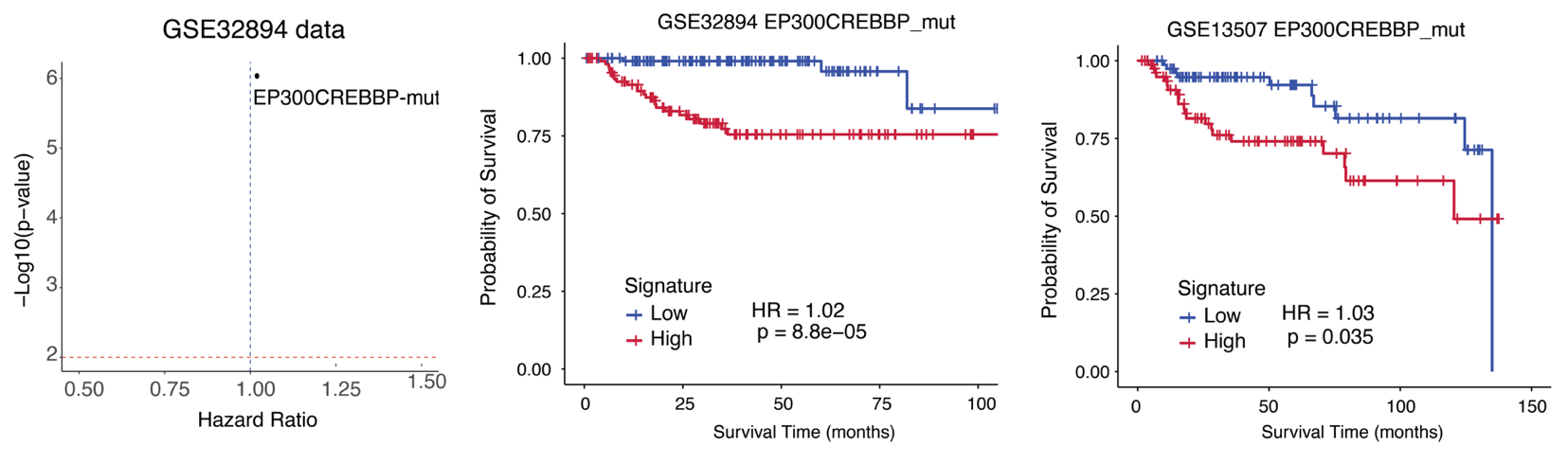


**Figure SF2:** Prognostic prediction of combination of CREBBP mutation status with EP300 mutation and developed signature scores of EP300CREBBP-mut.

**Figure SF3:** GSE32894 dataset:A. Boxplots explains the signature score of CREBBP-mut can differentiate into different tumor stages in GSE32894 datasets (where non-MI: Ta and T1, T2 and T3 stages). B. Survival analysis of patients with the molecular subtypes.

**Figure SF4:** Correlation of epiRG aberrations signature score with Immune pathways (SCC). Intensity of red color represents the strong positive correlation, and blue color indicates the negative correlation.


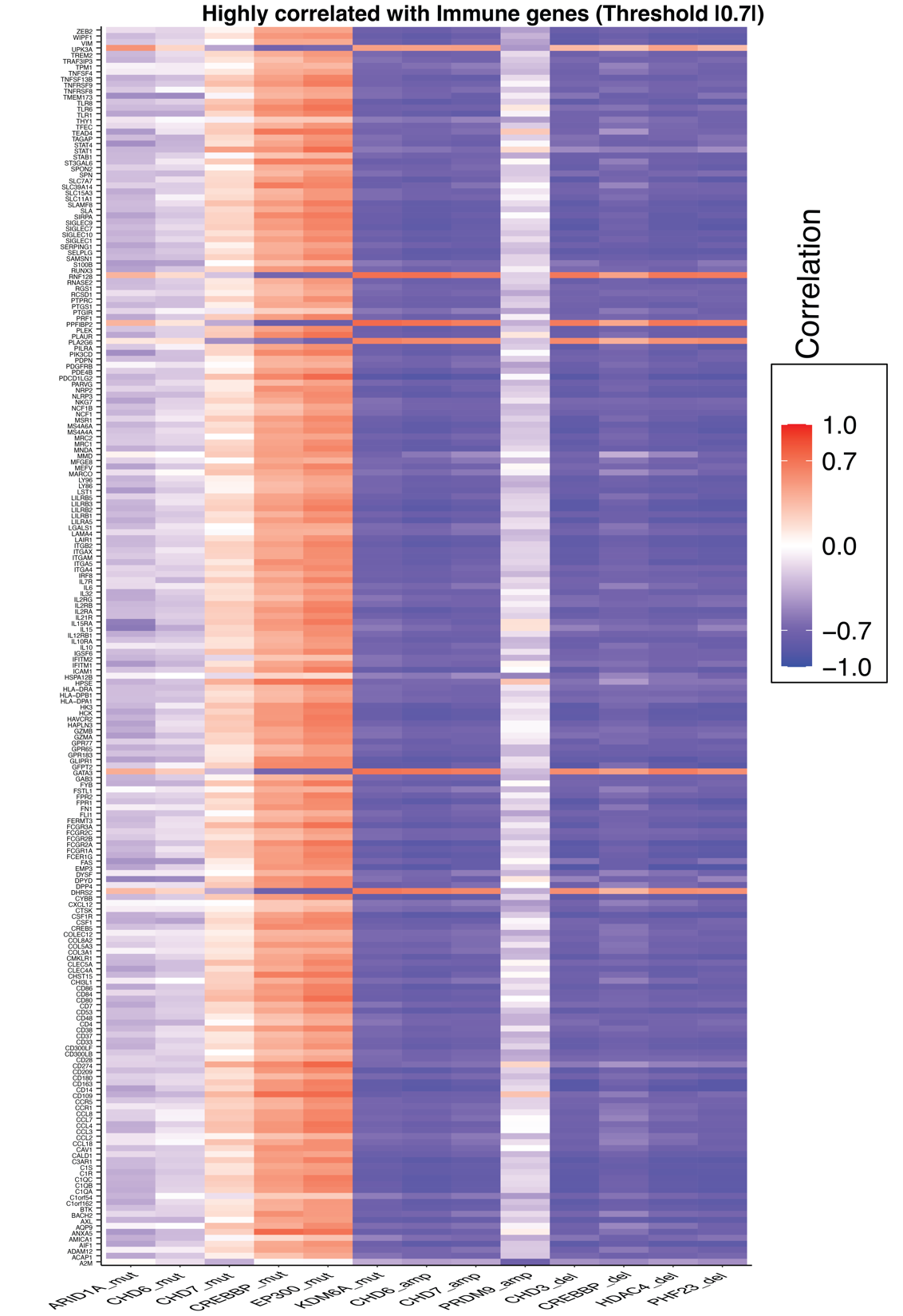


**Figure SF5:** Highly correlation of epiG-aber signature scores with immune genes (|SCC|≥ 0.7).

B-cells:

- Highly correlation signatures with B-cell marker genes, including CD38, CD20 (MS4A1), CD19, CD79, CD24, CD21 (CD2), CD1D, CD86, CD40, and CD138. Among the listed markers, **CD40** plays a role in reducing B.

CD8 T Cells:

- CD8A: A marker for cytotoxic T cells.
- CD8B: Another subunit of the CD8 molecule.

CD4 T Cells:

- CD4: Expressed on helper T cells and plays a crucial role in immune responses.
- CD3D and CD3E: Part of the T cell receptor complex.
- CD19 and CD79A: B cell markers, but also expressed on some T cells.

Macrophages:

- CD68: A common marker for macrophages.
- CD163: Expressed on anti-inflammatory macrophages 2.

Dendritic Cells (DCs):

- CLEC4C, LILRB4, NRP1, CCR7, B220 (in mice), and SiglecH (in mice) are markers for different DC subsets.

Neutrophils:

- Neutrophils play a dual role in immunity: they can both activate and suppress immune responses.
- While there isn’t a single specific marker for neutrophils, some commonly used genes include PTPRC, CD14, CD68, and MME

Additionally, we observed interactions with several genes that play critical roles in bladder cancer. Specifically, UPK3A, RNF128, PPFIBP2, PLA2G6, GATA3, and DHRS2 demonstrate negative correlations with CREBBP-mut, EP300-mut, CHD7-mut, and PRDM9-amp, while all remaining genes show positive correlations. Notably, UPK3A has emerged as a promising novel urinary marker in bladder cancer (13), and downregulation of RNF128 is associated with poor prognosis (14). PPFIBP2 serves as a biomarker for invasiveness and is closely linked to prognosis (15), while reduced expression or loss of GATA3 facilitates cell migration and invasion (16). Additionally, elevated DHRS2 expression correlates with prolonged survival (17).

**Table ST1:** Univariate Cox regression analysis for estimating the overall survival of bladder cancer patients using GSE13507 data.

| **Parameters** | **HR** | **95% CI (Lower and Upper)** | **P-value** |
| --- | --- | --- | --- |
| **Univariate** | | | |
| Signature score CREBBP-mut | 1.48 | 1.1 - 2 | 0.0096 |
| Age | 1.04 | 1.00 - 1.08 | 0.02 |
| Invasiveness (NMIBC vs MIBC) | 6.1 | 2.8 - 13.1 | 3.54e-06 |
| Tumor Stage  Ta (Reference) vs T1 | 1.25 | 0.31 -4.9 | 0.75 |
| T2 | 4.15 | 0.99 - 17.35 | 0.05 |
| T3 | 11 | 2.73 - 43.3 | 0.0006 |
| T4 | 26 | 4.36 -155.6 | 0.0003 |

**Table ST2:** Multivariate Cox regression analysis for estimating the overall survival of bladder cancer patients using Sjodahl GSE32894 data.

| Model-1 | **coef** | | **exp(coef)** | **se(coef)** | **z** | **p** | **lower_95** | **upper_95** | **TERM** |
| --- | --- | --- | --- | --- | --- | --- | --- | --- | --- |
| **Signature score CREBBP-mut** | 0.5333 | | 1.7046 | 0.1916 | 2.7838 | 0.0054 | 1.171 | 2.4814 | CREBBP_mut (sig score) |
| **Pre-Early Stage** | Reference | | | | | | | | Ta |
| **Early Stage** | 2.1772 | | 8.8213 | 1.0944 | 1.9893 | 0.0467 | 1.0326 | 75.3591 | Early Stage (T1, T2) |
| **Late Stage** | 2.5069 | | 12.2667 | 1.2652 | 1.9815 | 0.0475 | 1.0275 | 146.4372 | Late Stage (T3, T4) |
| **urothelial_A** | Reference | | | | | | | | urothelial_A |
| **genomically unstable** | 0.486 | | 1.6258 | 0.8775 | 0.5539 | 0.5797 | 0.2912 | 9.0783 | genomically unstable |
| **infiltrated** | 0.125 | | 1.1331 | 0.995 | 0.1256 | 0.9 | 0.1612 | 7.9653 | infiltrated |
| **SCC-like** | 0.0779 | | 1.081 | 1.1182 | 0.0697 | 0.9444 | 0.1208 | 9.6754 | SCC-like |
| **urobasal B** | 2.0491 | | 7.7613 | 0.8852 | 2.3148 | 0.0206 | 1.369 | 44.0009 | urobasal B |
| Model-2 | | | | | | | | | |
| **CREBBP_mut** | | 0.5898 | 1.8036 | 0.1733 | 3.4033 | 7E-04 | 1.2842 | 2.5331 | CREBBP_mut (sig score) |
| **urothelial_A** | | Reference | | | | | | | urothelial_A |
| **genomically unstable** | | 1.5819 | 4.8641 | 0.828 | 1.9105 | 0.0561 | 0.9598 | 24.6491 | genomically unstable |
| **infiltrated** | | 0.9176 | 2.5034 | 0.9556 | 0.9603 | 0.3369 | 0.3847 | 16.2909 | infiltrated |
| **SCC-like** | | 1.0871 | 2.9655 | 1.0907 | 0.9967 | 0.3189 | 0.3497 | 25.149 | SCC-like |
| **urobasal B** | | 2.8748 | 17.7211 | 0.8519 | 3.3745 | 7E-04 | 3.3368 | 94.1146 | urobasal B |

**Table ST3:** Correlation analysis with tumor-infiltrating lymphocytes (TILs) of six immune cell types from TIMER data.

| **epiRG-aber** | **B-cells** | | **CD4-Tcell** | | **CD8-Tcell** | | **Neutrophil** | | **Macrophage** | | **Dendritic** | |
| --- | --- | --- | --- | --- | --- | --- | --- | --- | --- | --- | --- | --- |
|  | **SCC** | **P-value** | **SCC** | **P-value** | **SCC** | **P-value** | **SCC** | **P-value** | **SCC** | **P-value** | **SCC** | **P-value** |
| **ARID1A-mut** | 0.1273 | 0.010 | -0.3681 | 1.65E-14 | -0.1607 | 0.001 | -0.3361 | 4.46E-12 | 0.1161 | 0.019 | -0.3944 | 0 |
| **CHD6-mut** | 0.0361 | 0.467 | -0.3193 | 4.272E-11 | -0.2194 | 7.92E-06 | -0.3843 | 5.80E-16 | -0.0866 | 0.081 | -0.3216 | 3.90E-11 |
| **CHD7-mut** | -0.0898 | 0.070 | 0.0449 | 0.366 | 0.3012 | 5.563E-10 | 0.2817 | 8.48E-09 | 0.0855 | 0.085 | 0.331 | 9.73E-12 |
| **CREBBP-mut** | -0.16 | 0.001 | 0.2971 | 9.68E-10 | 0.5559 | 2.22E-34 | 0.5176 | 0 | 0.2277 | 3.48E-06 | 0.6507 | 0 |
| **EP300-mut** | -0.1249 | 0.011 | 0.3512 | 2.95E-13 | 0.6052 | 5.03E-42 | 0.6811 | 0 | 0.229 | 3.04E-06 | 0.7756 | 0 |
| **KDM6A-mut** | 0.1306 | 0.008 | -0.4952 | 1.44E-26 | -0.6243 | 2.31E-45 | -0.7588 | 0 | -0.4837 | 2.92E-25 | -0.8038 | 0 |
| **CHD6-amp** | 0.1426 | 0.003 | -0.5297 | 8.17E-31 | -0.5915 | 9.18E-40 | -0.7873 | 0 | -0.4165 | 1.65E-18 | -0.8225 | 0 |
| **CHD7-amp** | 0.1186 | 0.016 | -0.5906 | 1.24E-39 | -0.5662 | 7.18E-36 | -0.8218 | 0 | -0.3886 | 4.02E-16 | -0.8195 | 0 |
| **PRDM9-amp** | -0.1201 | 0.015 | -0.1517 | 0.002 | 0.1447 | 0.003 | 0.0015 | 0.97 | -0.2089 | 2.16E-05 | 0.1069 | 0.031 |
| **CHD3-del** | 0.1344 | 0.006 | -0.4949 | 1.54E-26 | -0.518 | 2.57E-29 | -0.7036 | 0 | -0.496 | 1.15E-26 | -0.77 | 0 |
| **CREBBP-del** | 0.1052 | 0.033 | -0.5998 | 3.98E-41 | -0.4327 | 5.33E-20 | -0.7539 | 0 | -0.3277 | 1.20E-11 | -0.7043 | 0 |
| **HDAC4-del** | 0.1689 | 0.0006 | -0.5712 | 1.30E-36 | -0.5312 | 5.20E-31 | -0.7529 | 0 | -0.3929 | 1.79E-16 | -0.7889 | 0 |
| **PHF23-del** | 0.1301 | 0.008 | -0.4927 | 2.80E-26 | -0.5124 | 1.25E-28 | -0.7001 | 0 | -0.5003 | 3.59E-27 | -0.7628 | 0 |

**Table ST4:** Global differential methylation analysis of bladder cancer patients with aberration to wild type

| **Genomic aberration** | **Total DMCpG’s** | **Number of up DMCpG’s** | **Number of down DMCpG’s** | **Percentage Upregulated** | **Percentage Downregulated** | **Deviation Percentage** |
| --- | --- | --- | --- | --- | --- | --- |
| ARID1A-mut | 54 | 51 | 3 | 94.44 | 5.56 | 88.89 |
| CHD6-mut | 510 | 234 | 276 | 45.88 | 54.12 | 8.24 |
| CHD7-mut | 215 | 79 | 136 | 36.74 | 63.26 | 26.51 |
| CREBBP-mut | 119 | 5 | 114 | 4.20 | 95.80 | 91.60 |
| EP300-mut | 134 | 8 | 126 | 5.97 | 94.03 | 88.06 |
| **KDM6A-mut** | **8755** | **231** | **8524** | **2.64** | **97.36** | **94.72** |
| CHD6-amp | 19828 | 7924 | 11904 | 39.96 | 60.04 | 20.07 |
| CHD7-amp | 19863 | 3844 | 16019 | 19.35 | 80.65 | 61.29 |
| PRDM9-amp | 5277 | 3539 | 1738 | 67.06 | 32.94 | 34.13 |
| CHD3-del | 37602 | 7304 | 30298 | 19.42 | 80.58 | 61.15 |
| CREBBP-del | 24735 | 5039 | 19696 | 20.37 | 79.63 | 59.26 |
| HDAC4-del | 55383 | 13069 | 42314 | 23.60 | 76.40 | 52.81 |
| PHF23-del | 40839 | 7549 | 33290 | 18.48 | 81.52 | 63.03 |

DMCpG’s: differential methylated CpG’s

1. Alsaab HO, Sau S, Alzhrani R, Tatiparti K, Bhise K, Kashaw SK, et al. PD-1 and PD-L1 Checkpoint Signaling Inhibition for Cancer Immunotherapy: Mechanism, Combinations, and Clinical Outcome. Front Pharmacol. 2017;8:561.

2. Li T, Fu J, Zeng Z, Cohen D, Li J, Chen Q, et al. TIMER2.0 for analysis of tumor-infiltrating immune cells. Nucleic Acids Res. 2020;48(W1):W509-W14.

3. Fang S, Xu T, Xiong M, Zhou X, Wang Y, Haydu LE, et al. Role of Immune Response, Inflammation, and Tumor Immune Response-Related Cytokines/Chemokines in Melanoma Progression. J Invest Dermatol. 2019;139(11):2352-8.e3.

4. Castanza AS, Recla JM, Eby D, Thorvaldsdóttir H, Bult CJ, Mesirov JP. Extending support for mouse data in the Molecular Signatures Database (MSigDB). Nat Methods. 2023;20(11):1619-20.

5. Hänzelmann S, Castelo R, Guinney J. GSVA: gene set variation analysis for microarray and RNA-seq data. BMC Bioinformatics. 2013;14:7.

6. Charoentong P, Finotello F, Angelova M, Mayer C, Efremova M, Rieder D, et al. Pan-cancer Immunogenomic Analyses Reveal Genotype-Immunophenotype Relationships and Predictors of Response to Checkpoint Blockade. Cell Rep. 2017;18(1):248-62.

7. Bindea G, Mlecnik B, Tosolini M, Kirilovsky A, Waldner M, Obenauf AC, et al. Spatiotemporal dynamics of intratumoral immune cells reveal the immune landscape in human cancer. Immunity. 2013;39(4):782-95.

8. Xu L, Deng C, Pang B, Zhang X, Liu W, Liao G, et al. TIP: A Web Server for Resolving Tumor Immunophenotype Profiling. Cancer Res. 2018;78(23):6575-80.

9. Schaafsma E, Jiang C, Nguyen T, Zhu K, Cheng C. Microglia-Based Gene Expression Signature Highly Associated with Prognosis in Low-Grade Glioma. Cancers (Basel). 2022;14(19).

10. Nguyen TT, Lee HS, Burt BM, Amos CI, Cheng C. A combination of intrinsic and extrinsic features improves prognostic prediction in malignant pleural mesothelioma. Br J Cancer. 2022;127(9):1691-700.

11. Mariathasan S, Turley SJ, Nickles D, Castiglioni A, Yuen K, Wang Y, et al. TGFβ attenuates tumour response to PD-L1 blockade by contributing to exclusion of T cells. Nature. 2018;554(7693):544-8.

12. Price ME, Cotton AM, Lam LL, Farré P, Emberly E, Brown CJ, et al. Additional annotation enhances potential for biologically-relevant analysis of the Illumina Infinium HumanMethylation450 BeadChip array. Epigenetics Chromatin. 2013;6(1):4.

13. Lai Y, Ye J, Chen J, Zhang L, Wasi L, He Z, et al. UPK3A: a promising novel urinary marker for the detection of bladder cancer. Urology. 2010;76(2):514.e6-11.

14. Lee YY, Wang CT, Huang SK, Wu WJ, Huang CN, Li CC, et al. Downregulation of RNF128 Predicts Progression and Poor Prognosis in Patients with Urothelial Carcinoma of the Upper Tract and Urinary Bladder. J Cancer. 2016;7(15):2187-96.

15. He Y, Wu Y, Liu Z, Li B, Jiang N, Xu P, et al. Identification of Signature Genes Associated With Invasiveness and the Construction of a Prognostic Model That Predicts the Overall Survival of Bladder Cancer. Front Genet. 2021;12:694777.

16. Li Y, Ishiguro H, Kawahara T, Kashiwagi E, Izumi K, Miyamoto H. Loss of GATA3 in bladder cancer promotes cell migration and invasion. Cancer Biol Ther. 2014;15(4):428-35.

17. Xu F, Tang Q, Wang Y, Wang G, Qian K, Ju L, et al. Development and Validation of a Six-Gene Prognostic Signature for Bladder Cancer. Front Genet. 2021;12:758612.
